# Spatiotemporal differentiation of urban-rural income disparity and its driving force in the Yangtze River Economic Belt during 2000-2017

**DOI:** 10.1371/journal.pone.0245961

**Published:** 2021-02-04

**Authors:** Lingling Chen, Wei Shen

**Affiliations:** 1 Zhengzhou Tourism College, Zhengzhou, China; 2 College of Environment and Planning, Henan University, Kaifeng, China; 3 Key Laboratory of Geospatial Technology for Middle and Lower Yellow River Region, Henan University, Kaifeng, China; Northeastern University, CHINA

## Abstract

The income imbalance between urban and rural areas has seriously affected social fairness and justice and has become a key factor restricting the sustainable development of the economy and society. The analysis of the spatiotemporal laws and causes of urban-rural income disparity is of great significance to realizing the coordinated and integrated development of regional urban and rural areas. In this study, the coefficient of variation, Theil decomposition index, spatial autocorrelation method and GeoDetector model were used to analyze the spatiotemporal characteristics of the urban-rural income gap and its driving force in the Yangtze River Economic Belt from 2000 to 2017. The results show that the per capita disposable income of urban and rural residents in the study area shows a trend of rapid growth from 2000 to 2017. The urban-rural income gap in the study area showed an inverted "U"-shaped development process as a whole, and the relative difference showed an increasing trend. Regarding the spatial pattern, the study area showed a significant east-west differentiation pattern. The spatial distribution of the urban-rural income gap in the study area has an obvious positive spatial correlation, that is, the phenomena of high-value agglomeration and low-value agglomeration were significant. The economic development level, the industrial structure, the regional development policy, transportation, topographical conditions and resource endowments can strongly explain the spatial differentiation pattern of the urban-rural income gap in the study area. The spatial differentiation pattern of the urban-rural income gap is affected by both natural factors and socioeconomic factors. Among them, socioeconomic factors are the dominant factors, followed by natural factors. There is a significant interaction between natural factors and socio-economic factors, and the combination of socio-economic factors and adverse natural factors can significantly affect the regional urban-rural income gap.

## Introduction

Since the reform and opening up, China has implemented a series of urban-biased development policies under the guidance of unbalanced regional development strategies. Through long-term efforts, economic and social development has made remarkable achievements. With its advantageous geographical location and development policies, the regional social economy of the Yangtze River Economic Belt has developed rapidly, and the income of residents has also been significantly improved. However, in sharp contrast to the rapid economic growth, the urban-rural income disparity in the Yangtze River Economic Belt shows a widening trend. A relatively fair income distribution can provide a stable external environment for regional social and economic development; however, the growing material needs of residents in the Yangtze River Economic Belt make the income gap between the rich and the poor increasingly prominent. The widening urban-rural income disparity runs counter to the construction of a harmonious society since is not conducive to the improvement of the production enthusiasm of low-income people, which will affect the production efficiency of the entire society [1]. Under the background of social overcapacity and insufficient domestic demand, the urban-rural income disparity further aggravates these problems. The income imbalance between urban and rural areas has seriously affected social fairness and justice and has become a key factor restricting the sustainable development of the economy and society. Therefore, analyzing the causes of the urban-rural income disparity in the Yangtze River Economic Belt and effectively curbing the income gap between urban and rural areas is of great significance for building a sustainably developing society.

The urban-rural income disparity has been a hot topic in academic research, and the existing research can be divided into theoretical research and empirical research. Regarding the theoretical research, Kuznets first proposed the inverted U-shaped curve hypothesis of the relationship between economic development and the change in the income gap [2]. Robinson [3] and Ahluwalia [4] demonstrated the inevitability of the existence of inverted U-shaped curve of the income gap. Subsequently, related theories such as dual economic structure theory, unequal opportunity theory, human capital theory, income redistribution theory, the Fela model, regional network theory, urban-rural interaction and others have been proposed [5–9]. Empirical research includs the calculation of urban-rural income gap [10–13], the evolutionary process, the spatiotemporal evolution of the urban-rural relationship, and governance experience [10, 11]. Empirical research also assesses the relationship between urban-rural income gap and economic growth [14], the industrial structure [15], industrialization [16], urbanization [17, 18], financial development [19], foreign trade [20], investment [20], fiscal expenditures [18], and labor mobility [21], which are the influencing factors of the income gap between urban and rural areas [10, 22]. The selection of indicators includes not only the absolute and relative indexes of the urban-rural income disparity, but also the compound indicators such as the difference in the cost of living between urban and rural areas and all kinds of hidden subsidies, labor migration, environmental pollution, urban and rural public services, and other. Because the urban-rural income ratio can directly reflect the quality of life and the economic and social structure of urban and rural residents to a large extent, it has become the most commonly used index to study the income gap between urban and rural areas [10, 11]. Generally, scholars have made many meaningful explorations conducting theoretical and empirical research of the urban-rural income gap. However, there are still some deficiencies in the following aspects. First, there is a lack of comprehensive analysis of the temporal and spatial evolution of the income gap between urban and rural areas under a long time series. Second, the analysis of the influencing factors takes into account many social and economic factors, such as economic development, urbanization, the regional development policy of industrialization, the dual economic system, education levels, population size, transportation, economic extroversion, financial development, agricultural investment and others. However, there is a lack of analysis of natural environmental factors. Third, there is a lack of research on the interaction between factors, because the income of urban and rural residents is affected not only by the comprehensive quality of "people", but also by many factors such as socioeconomic factors and natural factors, which make the disparity systematic problem.

The Yangtze River Economic Belt is an economic belt with the largest population and the fastest social and economic development in China. It is also an inland river economic belt with global influence. This study uses the Yangtze River Economic Belt as the typical study are. The research is conducted as follows: First, this paper analyzes the spatiotemporal differentiation characteristics of the income levels of urban and rural residents in the study area based on the panel data of 130 cities in the Yangtze River Economic Belt from 2000 to 2017. Second, we analyze the spatiotemporal differentiation characteristics of the urban-rural income disparity and the comprehensive characteristics of its types. Finally, we integrate multidisciplinary knowledge such as economics, sociology and geography to build an expanded index system of the influencing factors of the urban-rural income gap. Then, we use the Geodetector model to analyze the main influencing factors of the urban-rural income gap in the study area, as well as the interaction of factors, in order to provide a theoretical reference for relevant departments to formulate differentiated development policies.

## Study area and data sources

### Study area

**Fig 1:**
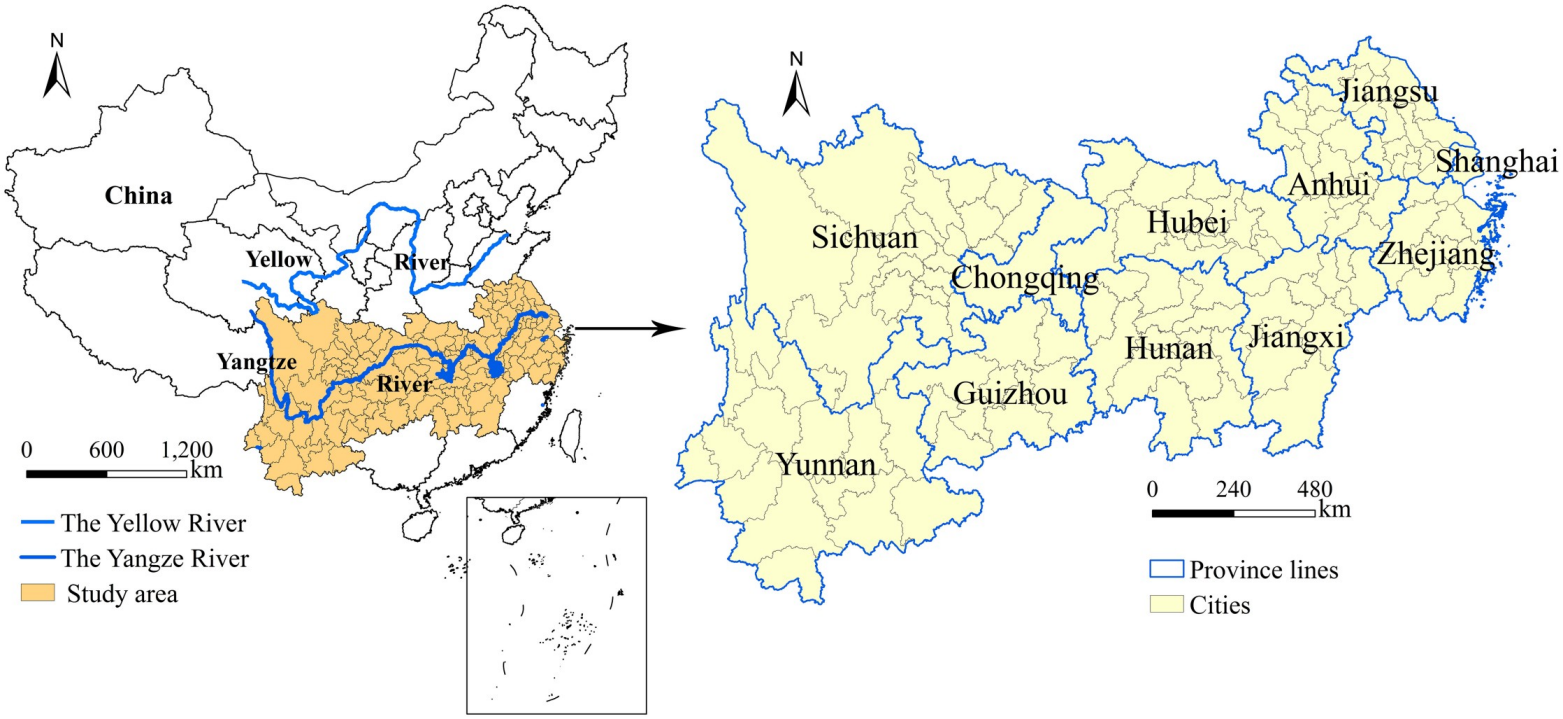
The location of the Yangtze River Economic Belt in China and an overview of the study area.

The Yangtze River Economic Belt spans the eastern, central and western regions of China, covering 11 provinces and cities, including Shanghai, Jiangsu, Zhejiang, Anhui, Jiangxi, Hubei, Hunan, Chongqing, Guizhou, Sichuan and Yunnan, with a total area of approximately 2.05 million square kilometers (Fig 1). The population and GDP of the Yangtze River Economic Belt are more than 40% of China’s totals. It is the river basin economic belt with the largest population, the largest industrial scale and the most complete urban system in the world. Through the golden waterway of the Yangtze River, the Yangtze River Economic Belt naturally connects the east (Shanghai, Jiangsu, Zhejiang, and Anhui), the middle (Jiangxi, Hubei, and Hunan) and the west (Chongqing, Guizhou, Sichuan, and Yunnan). It is connected with the Silk Road Economic Belt to the west, forming a new situation of opening up and playing an extremely important role in the national economic development strategy. Based on this, this study takes the Yangtze River Economic Belt in China as the study area and explores the spatiotemporal differentiation characteristics and driving force of the urban-rural income gap, in order to provide some reference for the coordinated development of urban-rural areas in the Yangtze River Economic Belt.

### Data sources

In this study, the per capita disposable income of urban and rural residents and the socioeconomic indicators in 130 cities of the Yangtze River Bconomic Belt from 2000 to 2017 are derived from provincial and municipal statistical yearbooks and socioeconomic statistical bulletins. The raster data of net primary productivity (NPP), temperature, precipitation and elevation are provided by the Data Center for Resources and Environmental Sciences, Chinese Academy of Sciences (RESDC) (http://www.resdc.cn) and Geographical Information Monitoring Cloud Platform (http://www.dsac.cn), and the spatial resolution is 1km. The vector boundary data of the Yangtze River Economic Belt are derived from the National Science and Technology Basic Condition Platform of China—National Earth System Science Data Center (http://www.geodata.cn).

## Methods

### Variation coefficient and Theil index

The variation coefficient is used to reflect the degree of discretization of the data, and it is an important index to measure the regional differences between years. The Theil index can measure the relative differences in regional economic development. In this paper, the variation coefficient and Theil index are used to measure the regional differences of the urban-rural income disparity in the Yangtze River Economic Belt. The specific calculation formula is as follows:

$$CV=\frac{1}{\overset{-}{y}}\sqrt{\frac{\sum_{i=1}^{n}{\left(y_{i}-\overset{-}{y}\right)}^{2}}{n}}$$ (1)

$$T=\frac{1}{n}\sum_{i=1}^{n}\frac{y_{i}}{\overset{-}{y}}log(\frac{y_{i}}{\overset{-}{y}})$$ (2)

In formulas (1) and (2), *CV* is the variation coefficient, T is the Theil index, n is the number of research units, *y*_*i*_ is the urban-rural income disparity of city *I*, and $\overset{-}{y}$ is the average value of the urban-rural income disparity in all cities. The smaller the variation coefficient and Theil index are, the smaller the regional difference.

The Theil decomposition index can decompose the overall differences into differences within and between sub-regions. In order to prevent the difference from being offset when the Theil index is negative, the improved Theil decomposition index is used to analyze the difference between regions. The specific calculation formula is as follows [23–25]:

$$Th\text{eil}=\sum_{i=1}^{n}R_{i}\;\text{ln}(nR_{i})=T_{W}+T_{B}$$ (3)

$$T_{W}=\sum_{k=1}^{K}R_{k}|\sum_{i\in g_{k}}\frac{R_{i}}{R_{k}}\text{log}(\frac{R_{i}/R_{k}}{1/n_{k}})|$$ (4)

$$T_{B}=\sum_{k=1}^{K}R_{k}|\text{log}(R_{k}\frac{n}{n_{k}})|$$ (5)

In formulas (3)–(5), *T*_*W*_ is the difference among the east, middle and west of the study area; *T*_*B*_ is the difference among the east, middle and west; n is the number of cities; K represents the number of groups, that is, the east, middle and west; *g*_*k*_ represents the k group; *n*_*k*_ represents the number of samples in each group; and *R*_*i*_ is the ratio of the ith city to the study area. *R*_*k*_ is the ratio of the kth group to the study area.

### Spatial autocorrelation analysis method

We use the spatial autocorrelation analysis method to analyze whether there is a spatial agglomeration effect between urban-rural income gap variables. Spatial autocorrelation refers to the interdependence of spatial unit attributes in space, and is a way to measure the degree of spatial agglomeration [22, 26, 27]. According to the size of the spatial range, spatial autocorrelation can be divided into global spatial autocorrelation and local spatial autocorrelation. Global spatial autocorrelation measures the degree of spatial correlation of a region as a whole, and it is often measured by *Moran’s I* index. The global autocorrelation formula is:

$$I=\frac{n}{s_{o}}\frac{\sum_{i=1}^{n}\sum_{j=1}^{n}w_{ij}(x_{i}-\overset{-}{x})(x_{j}-\overset{-}{x})}{\sum_{i=1}^{n}{\left(x_{i}-\overset{-}{x}\right)}^{2}}$$ (6)

In formula (6), n is the total number of spatial units; *x*_*i*_ and *x*_*j*_ are the observed values of spatial units *i* and *j*, respectively; *w*_*ij*_ is the spatial weight matrix, which is used to reveal the spatial relations among the elements; and *s*_*o*_ is the aggregate of all the spatial weights, $s_{o}=\sum_{i=1}^{n}\sum_{j=1}^{n}w_{ij}$. The range of *Moran’s I* index is [-1,1].

Although global spatial autocorrelation can measure the overall spatial dependence of the observed variables, it ignores the local instability of the space to a certain extent. Therefore, in order to further explore the local spatial correlation features, the local spatial autocorrelation method is introduced to analyze the local spatial autocorrelation features. The specific calculation formula is as follows:

$$I_{i}=Z_{i}\sum_{i=1}^{n}w_{ij}Z_{j}$$ (7)

In formula (7), *Z*_*i*_ and *Z*_*j*_ are the standardized values of spatial units *i* and *j*, respectively, and *w*_*ij*_ is a spatial weight matrix, which is used to reveal the spatial relationship between spatial units.

### The GeoDetector method

GeoDetector represent a new spatial statistics method that is used to detect spatial heterogeneity and identify driving factors based on risk, factors, ecology, and interaction [28]. GeoDetector method can be divided into the factor detector, the interaction detector, the ecological detector and the risk detector. This method overcomes the limitations of many assumptions and large amounts of data found in the traditional mathematical statistical model [29].

#### Factor detector

We use the factor detector to analyze the main factors affecting the spatial differentiation of the urban-rural income gap in the Yangtze River Economic Belt. The specific model formula is as follows:

$$q=1-[\frac{\sum_{h=1}^{L}\sum_{i=1}^{N_{h}}{\left(Y_{hi}-\overset{-}{Y_{h}}\right)}^{2}}{\sum_{i=1}^{N}{\left(Y_{i}-\overset{-}{Y}\right)}^{2}}]=1-\frac{\sum_{h=1}^{L}N_{h}{\sigma_{h}}^{2}}{N\sigma^{2}}=1-\frac{SSW}{SST}$$ (8)

$$SSW=\sum_{h=1}^{L}\sum_{i=1}^{N_{h}}{\left(Y_{hi}-\overset{-}{Y_{h}}\right)}^{2}=\sum_{h=1}^{L}N_{h}{\sigma_{h}}^{2}$$ (9)

$$SST=\sum_{i=1}^{N}{\left(Y_{i}-\overset{-}{Y}\right)}^{2}=N\sigma^{2}$$ (10)

In formulas (8)–(10), *q* indicates the explanatory power of a factor to the urban-rural income gap. *h* is the number of layers of the influencing factor; *N*_*h*_ and *N* are layer *h* of the influence factor and the number of samples of the whole study area, respectively; *σ*_*h*_ and *σ* are the variance of the urban-rural income gap of layer h and the whole study area, respectively; *Y*_*i*_ is the ratio of urban to rural income in city *i*; *Y*_*hi*_ is the urban-rural income ratio of city *i* in layer *h*; *Y*_*h*_ is the average of all the cities in the layer *h*; *SSW* is the sum of the in-layer variances; and *SST* is the total variance of the study area. The range of *q* is [0, 1]; and the higher the *q* is, the stronger the explanatory power of this factor for the urban-rural income gap.

#### Interaction detector

The interaction detector is used to quantitatively identify the interactive influence of the interaction between factors on the urban-rural income gap, that is, to evaluate the level of influence of the combined effect (enhanced or weakened) on the urban-rural income gap.

#### Ecological detector

The ecological detector can analyze whether there is a significant difference in the influence of any two factors on the spatial distribution of the urban-rural income gap. The formula is as follows:

$$F=\frac{N_{X_{1}}(N_{X_{2}}-1)\times {SSW}_{X_{1}}}{N_{X_{2}}(N_{X_{1}}-1)\times {SSW}_{X_{2}}}$$ (11)

$${SSW}_{X_{1}}=\sum_{h=1}^{L_{1}}N_{h}{\sigma_{h}}^{2}$$ (12)

$${SSW}_{X_{2}}=\sum_{h=1}^{L_{2}}N_{h}{\sigma_{h}}^{2}$$ (13)

In formulas (12)–(14), $N_{X_{1}}$ and $N_{X_{2}}$ denote the sample quantities of factors *X*_1_ and *X*_2_, respectively; ${SSW}_{X_{1}}$ and ${SSW}_{X_{2}}$ represent the sum of the intralayer variances of the stratification formed by *X*_1_ and *X*_2_, respectively; and *L*_1_ and *L*_2_ are the number of layers of variables *X*_1_ and *X*_2_, respectively. The null hypothesis H0: ${SSW}_{X_{1}}={SSW}_{X_{2}}$. If *H*_*0*_ was rejected at the significance level *α*, a significant difference in the influence of factors *X*_1_ and *X*_2_ on the spatial distribution of the urban-rural income gap is significant.

### Selection and classification of influencing factors

In the long run, the improvement of the level of regional economic development will help to narrow the urban-rural income gap [30]. Li et al. found that the development of the secondary and tertiary industries in the Yangtze River Economic Belt can narrow the urban-rural income gap. In addition, an increase in the natural growth rate of the population will lead to an imbalance in the ratio of labor and capital and then affect the urban-rural income gap [1]. Traffic accessibility has a fundamental impact on the economic development of urban and rural areas and then has an important impact on the income of urban and rural residents [31]. Investment in fixed assets can have an impact on the urban-rural income gap by affecting the intensity of the resource development and market scale in urban and rural areas [31]. The natural environment is the basic condition of urban and rural construction and development and plays a fundamental role in the evolution of the urban-rural income gap. Among them, the terrain, geomorphology and climate and other environmental conditions affect the traffic, infrastructure construction and industrial development of urban and rural areas, thus indirectly affecting the income of urban and rural residents [32]. The natural resource endowment supports regional social and economic development and directly affecting the income of urban and rural residents [33].

Referring to relevant studies [34–36] and considering the availability of data on the research area, we set the urban-rural income gap of 130 cities in the Yangtze River Economic Belt as the dependent variable. Per capita GDP (X1), the urbanization rate (X2), the added value of the secondary industry share of GDP (X3), the added value of the tertiary industry of GDP (X4), per capita fixed asset investment (X5), the natural population growth rate (X6), the road network density (X7), the annual mean precipitation (X8), the annual mean temperature (X9), elevation (X10), the relief degree of land surface (X11), and NPP (X12) are set as explanatory variables (Table 1).

**Table 1:** Index system of influencing factors.

Influencing factor	Factors	Code	Indicators
**Socioeconomic factors**	Economic development level	X1	Per capitaGDP
	Urbanization level	X2	Urbanization rate
	Industrial structure	X3	The proportion of secondary industry in GDP
		X4	The proportion of tertiary industry in GDP
	Regional development policy	X5	Per capita fixed asset investment
	Population	X6	Natural population growth rate
	Traffic accessibility	X7	Road network density
**Natural environment factors**	Climatic conditions	X8	Annual mean precipitation
		X9	Annual mean temperature
	Topographic condition	X10	Elevation
		X11	Relief degree of land surface
	Resource endowment	X12	NPP

Calculation process of the RDLS: refer to related research [32]. RDLS is obtained by grid calculation. The specific calculation formula is as follows:

RDLS={[Max(H)−Min(H)]×[1−P(A)/A]}/500 (14)

In formula (14), RDLS is the relief degree of land surface, which is also known as the landform degree of relief. *Max*(H) and *Min*(H), which are the highest and lowest elevations in the region, respectively, are used to calculate the relative height difference. P (A) is the area of flat land (km^2^). In this paper, the area with a slope less than or equal to 2° is defined as flat land. A is the total area of the research unit.

Referring to the related research [29, 36, 37], we use the natural breakpoint method in ArcGIS10.3 software to divide X1-10 and X12 into six categories, and X11 into nine categories.

## Results

### Spatiotemporal characteristics of the disposable income of urban and rural residents

#### Temporal variation characteristics

The per capita disposable income of urban and rural residents in the Yangtze River Economic Belt showed a trend of rapid growth from 2000 to 2017. The absolute value of the per capita disposable income of urban residents increased from 6009 in 2000 to 33387 in 2017, an increase of nearly 5.56 times. The absolute value of the per capita disposable income of rural residents increased from 2339 in 2000 to 15056 in 2017, an increase of nearly 6.44 times.

We used the variation coefficient and Theil index to reflect the relative differences in the study area, the three subregions (east, middle and west), between regions and within regions. Regarding the relative differences of urban residents, the relative differences in the study area showed a trend of first increasing and then decreasing from 2000 to 2017 (S1 Table). Regarding the different regions, the relative differences in the eastern region showed a trend of fluctuating growth. The relative differences in the western and central regions both showed a development process of the "M" type, but the fluctuation range of the central region was smaller than that of the western region. Regarding between regions and within regions, the relative differences between regions showed a trend of narrowing, while the relative differences within regions showed a trend of expanding.

Regarding the relative differences of rural residents, the relative differences in the study area showed a trend of first increasing and then decreasing from 2000 to 2017 (S2 Table). The time variation trend of the relative differences in the western, central and eastern regions is the same, showing an inverted "U" shaped development process. Regarding between regions and within regions, the relative differences between regions and within regions show a development trend of first increasing and then decreasing.

#### Spatial pattern of the disposable income of urban and rural residents

**Fig 2:**
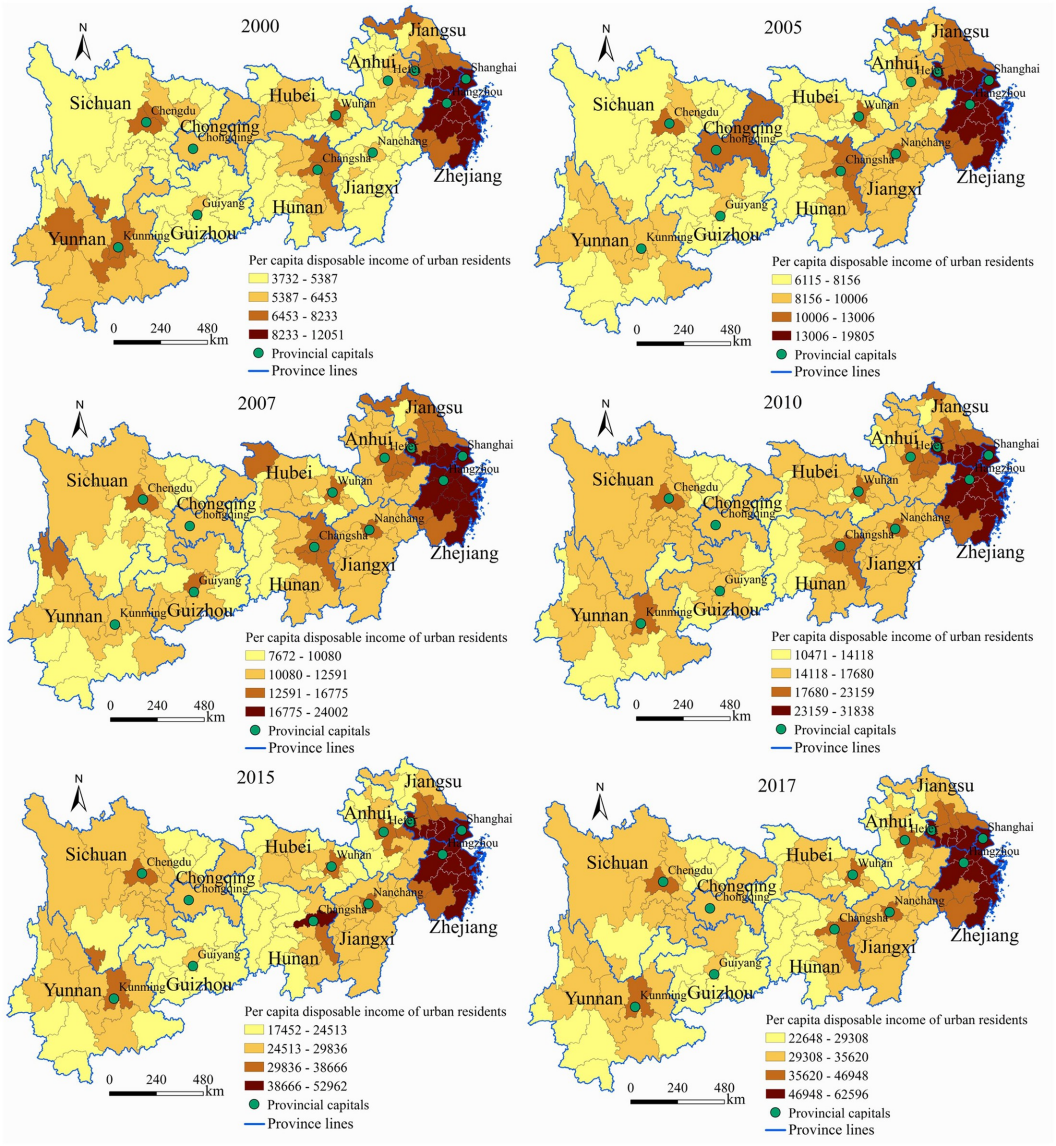
Spatial pattern evolution of per capita disposable income of urban residents in the Yangtze River Economic Belt from 2000 to 2017.

**Fig 3:**
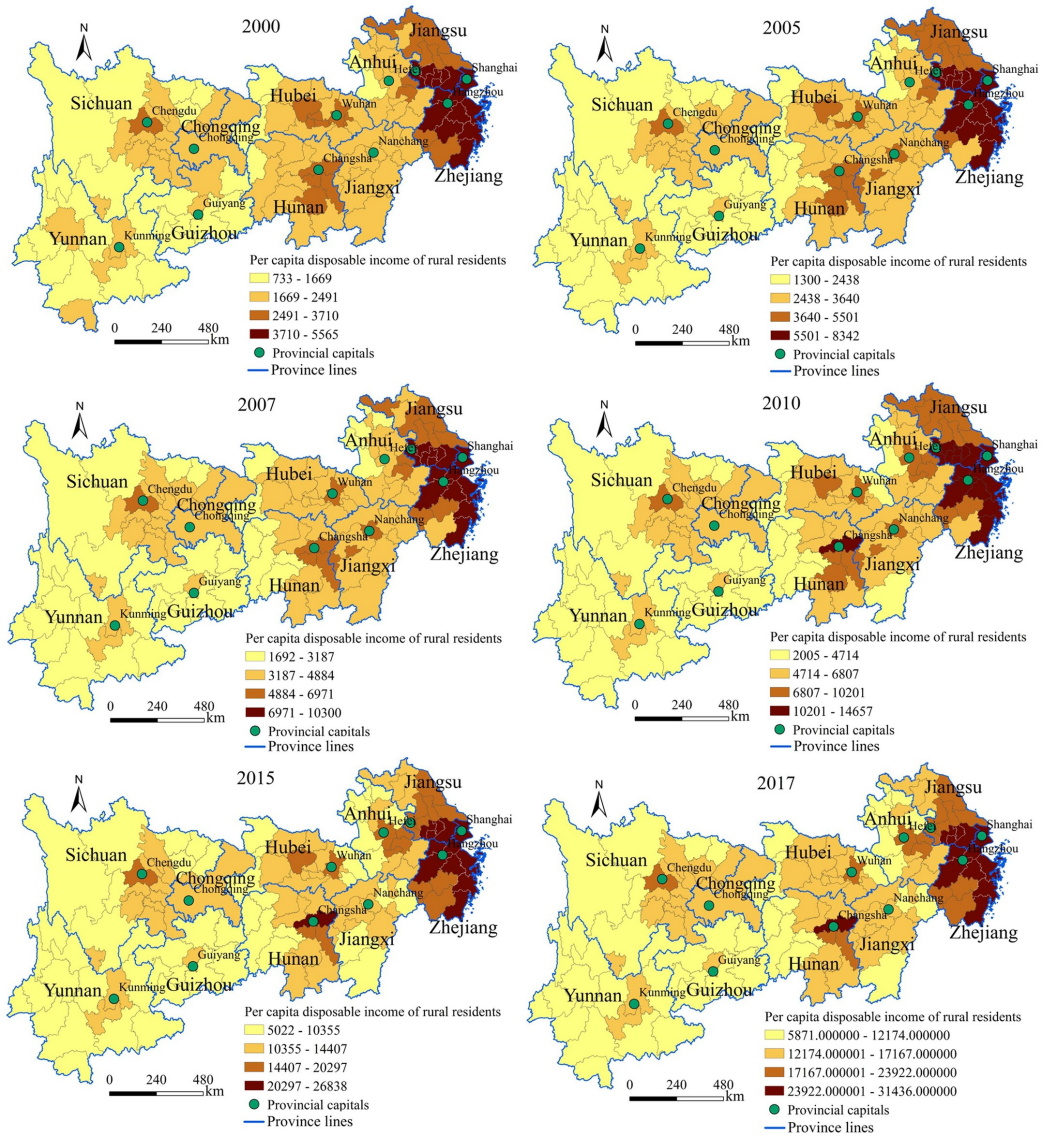
Spatial pattern evolution of the per capita disposable income of rural residents in the Yangtze River Economic Belt from 2000 to 2017.

By using the natural break point method in the ArcGIS10.3 software, the per capita disposable income of urban and rural residents in the Yangtze River Economic Belt from 2000 to 2017 is divided into four levels: low, medium, relatively high and high (Figs 2 and 3).

Regarding the spatial pattern of the per capita disposable income of urban residents, the high-income cities are mainly distributed in the Yangtze River Delta region (Fig 2). The higher-income cities are mainly provincial capitals. The middle-income cities are mainly distributed in the areas surrounding the provincial capitals. The low-income cities are mainly distributed in marginal areas with inconvenient transportation and underdeveloped economies, such as the Yunnan-Guizhou Plateau, western mountains of Sichuan Province, western Hunan Province, southern Jiangxi Province and northern Anhui Province. Regarding the evolutionary trend, the number of medium-income and high-income cities showed an inverted "U" shaped development trend.

Regarding the spatial pattern of the per capita disposable income of rural residents, the study area showed obvious echelon distribution characteristics (Fig 3). Among the regions, the overall income level of the eastern region was the highest, and most cities were at the higher and high income levels. Most cities in the central region were at the middle-income and higher-income levels and were mainly distributed in the provincial capital and its surrounding cities. The overall income level of the western region was the lowest, and most cities were at the low income level. Regarding the evolutionary trend, the overall change in the western region was relatively small while those in the central and eastern regions were relatively large.

### Spatiotemporal characteristics of urban-rural income disparity

#### Temporal variation characteristics

**Fig 4:**
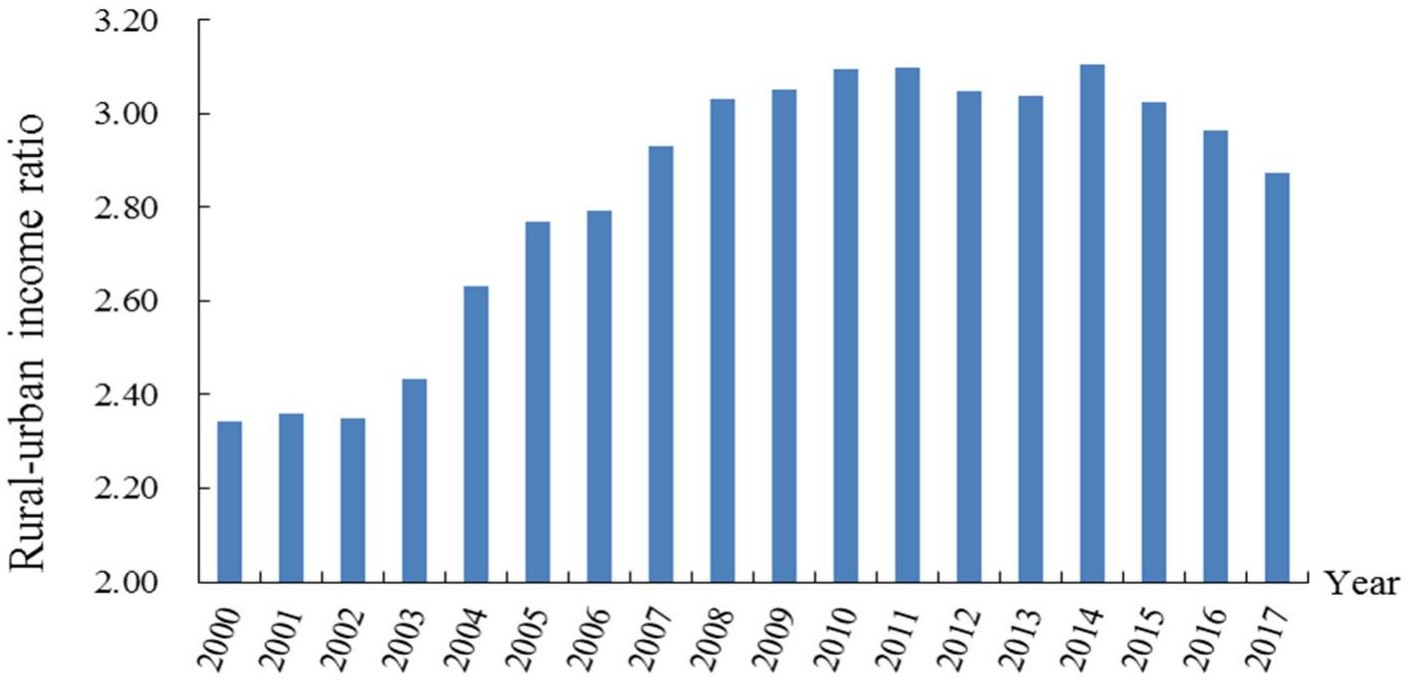
The evolution of the urban-rural income disparity in the study area from 2000 to 2017.

The urban-rural income disparity in the study area showed a trend of first increasing and then decreasing from 2000 to 2017 (Fig 4). The urban-rural income disparity was in expansionary stage from 2000 to 2011, slightly fluctuating from 2011 to 2014, and peaking in 2014. Then, in 2014–2017, the urban-rural income gap began to show a slow downward trend. The whole development process was consistent with Kuznets’ inverted U-shaped curve theory.

**Fig 5:**
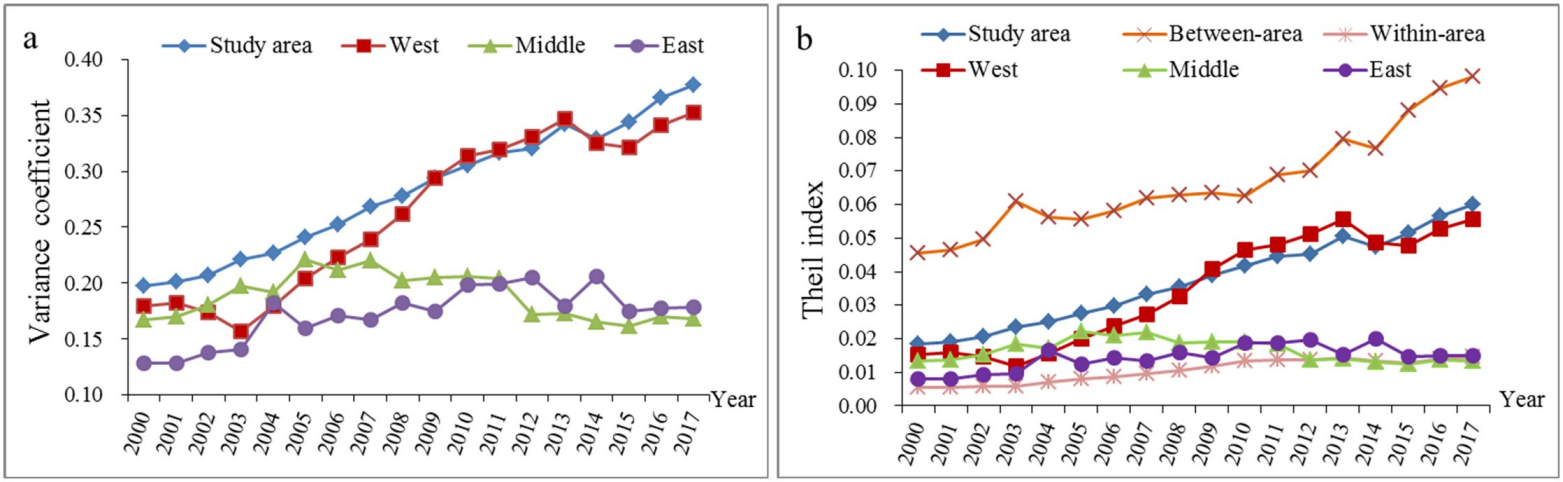
Regional differences in the urban-rural income disparity in the study area from 2000 to 2017.

Regarding the relative differences in the urban-rural income disparity, the relative difference in the study area showed a fluctuanting increasing trend from 2000 to 2017 (Fig 5). Regarding the different regions, the development trend of the western region was similar to that of the study area, while the relative differences in the central and eastern regions showed an inverted "U"-shaped trend. Regarding between regions and within regions, the relative differences between regions were much larger than those within regions, and there was a trend of further expansion in the study period.

#### Spatial pattern evolution

**Fig 6:**
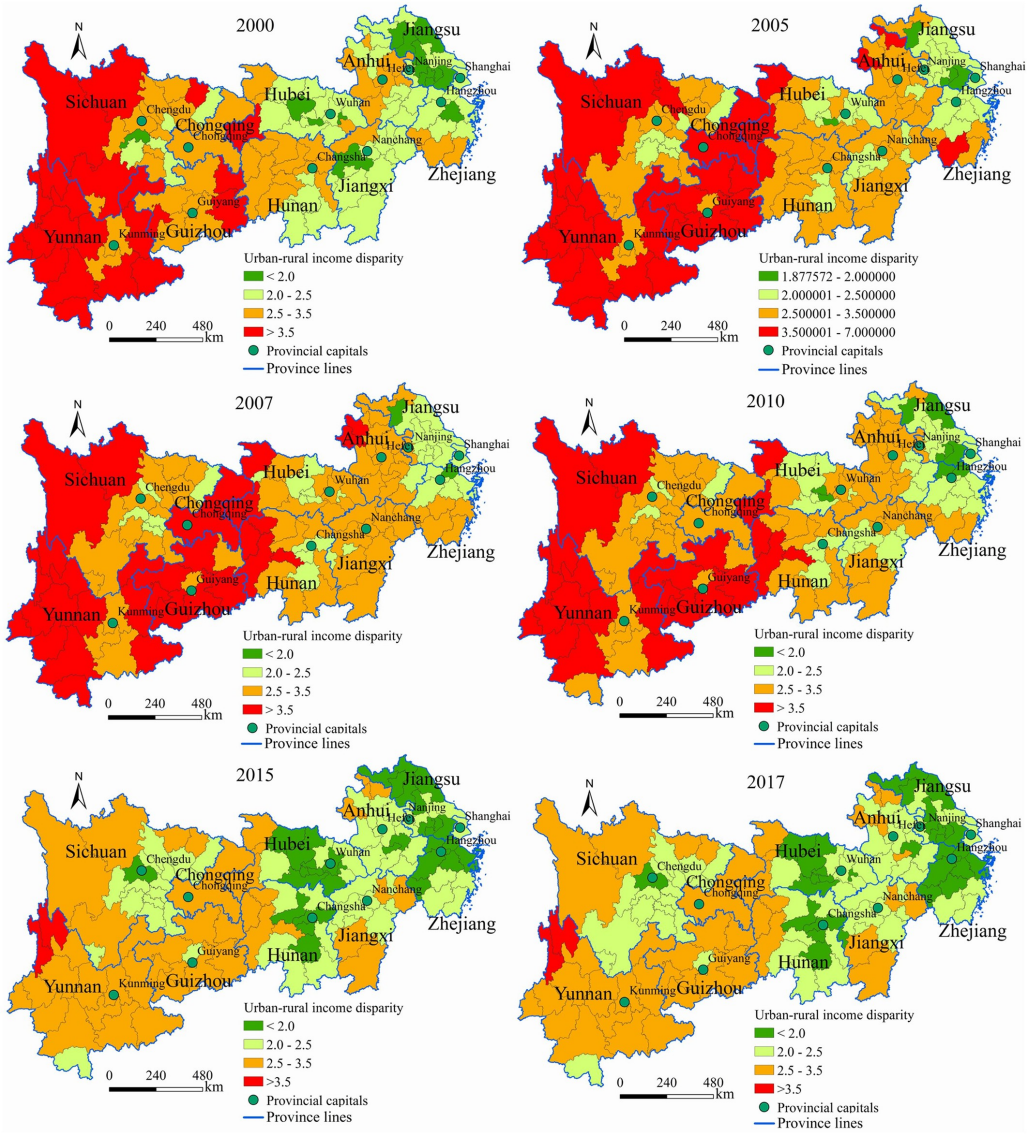
Spatial pattern evolution of the urban-rural income disparity in the study area from 2000 to 2017.

We divide the urban-rural income ratio into four categories: low urban-rural income gap (< 2), medium urban-rural income gap (2–2.5), higher urban-rural income gap (2.5–3.5), and high urban-rural income gap (> 3.5). The map of the evolution of the spatial patterns of the urban-rural income gap in the Yangtze River Economic Belt are obtained through spatial visualization (Fig 6).

The cities with high income gaps in the Yangtze River Economic Belt from 2000 to 2017 were mainly distributed in western Sichuan Province and the Yunnan-Guizhou Plateau. The cities with higher income gaps were mainly distributed in the Yunnan-Guizhou Plateau, the middle reaches, Anhui Province and southern Zhejiang. The cities with low income and middle income gaps were mainly distributed in the three central provinces and the Yangtze River Delta. The number of cities with high and higher income gaps first increased and then decreased while the number of cities with low and middle income gaps first decreased and then increased.

#### Spatial correlation effect of urban-rural income disparity

We used the spatial analysis tool in the GeoDa software to establish a spatial weight matrix and used global and local spatial autocorrelation methods to analyze the global and local spatial correlation features, respectively (Table 2). The results of the global spatial autocorrelation analysis show that the global *Moran’s I* index of the urban-rural income disparity was positive and passes the significance test. This shows that there is an obvious positive spatial correlation in the study area, that is, there are high-value agglomerations and low-value agglomerations.

**Table 2:** Analysis results of the global spatial autocorrelation.

Year	2000	2005	2007	2010	2015	2017
*Moran’s I*	0.624	0.554	0.519	0.584	0.644	0.626
*p-value*	0.001	0.001	0.001	0.001	0.001	0.001

**Fig 7:**
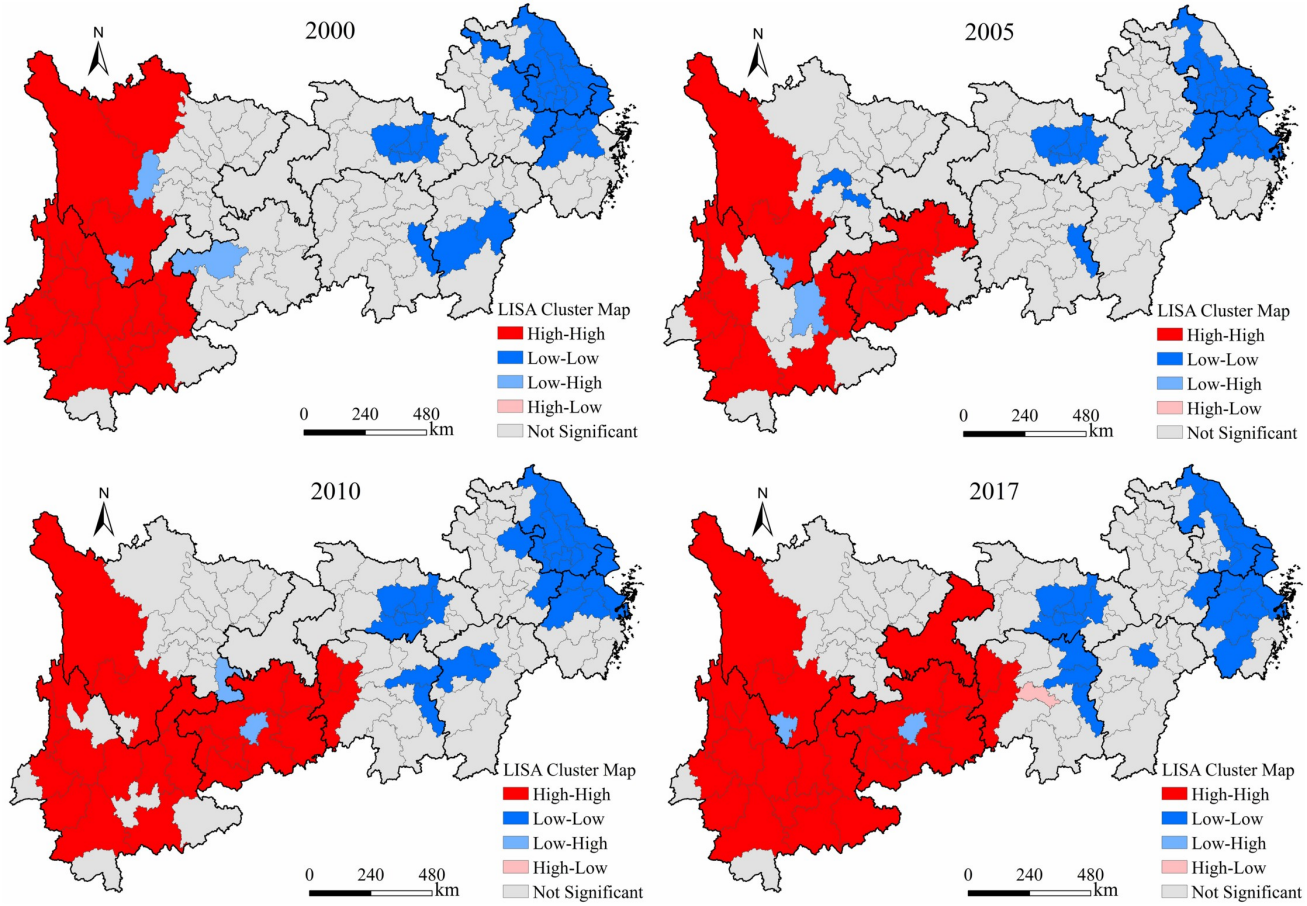
Local spatial autocorrelation of the urban-rural income disparity in the study area from 2000 to 2017.

Furthermore, we use the local spatial autocorrelation method to analyze the local spatial autocorrelation characteristics of the urban-rural income disparity in the Yangtze River Economic Belt. As shown in Fig 7, the high-high agglomeration areas were mainly distributed in Guizhou and Yunnan Provinces and mountainous cities in western Sichuan Province. Affected by the natural environment, traffic and other factors, the level of socioeconomic development in such areas was relatively low, and the spatial agglomeration of the urban-rural income disparity was prominent. The low-low agglomeration areas were distributed in Shanghai, Jiangsu Province, eastern Anhui Province, and the north-central part of Zhejiang Province. The low-high agglomeration area, including Guiyang, Panzhihua, Luzhou, Bijie and Ya’an city, was adjacent to the high-high agglomeration area in the west. During the research period, the high-high agglomeration area in the western study area tended to expand to the east, the low-low agglomeration area in the central study area tended to increase, and the low-low agglomeration area in the eastern study area tended to transfer to the eastern coastal areas.

### Driving forces of the spatial differentiation of the urban-rural income disparity

#### Geographical detection of driving factors

We use the factor detector of the GeoDetector model to analyze the influencing factors of the spatial differentiation of the urban-rural income disparity in the Yangtze River Economic Belt. As shown in Table 3, the *q* of each factor represents the explanatory power of the factor for the spatial differentiation of the urban-rural income gap. In 2015, the order of the explanatory power of the 12 factors is as follows: elevation (X10)> urbanization rate (X2) > road network density (X7) > per capita GDP (X1)> RDLS (X11) > the proportion of tertiary industry in GDP (X4) > per capita fixed asset investment (X5) > NPP (X12) > annual mean temperature (X9) > natural population growth rate (X6) > annual mean precipitation (X8) > the proportion of primary industry in GDP (X3).

**Table 3:** *q* and *p* values of the factors in 2015.

Factors	*q* value	*p* value
PerGDP (X1)	0.371	0.000
UR (X2)	0.512	0.000
SIOP (X3)	0.299	0.003
TIOP (X4)	0.072	0.651
PerFAI (X5)	0.271	0.005
NPGR (X6)	0.108	0.751
RND (X7)	0.397	0.000
AMP (X8)	0.086	0.248
AMT (X9)	0.168	0.972
DEM (X10)	0.532	0.000
RDLS (X11)	0.351	0.012
NPP (X12)	0.234	0.039

Regarding the socioeconomic factors, the economic development level, industrial structure, regional development policy and transportation all have strong explanatory power for the spatial differentiation of the urban-rural income gap, and the *p* values were all less than 0.01 (Table 3). Among the factors, the urbanization rate index (X2) has the greatest explanatory power for the urban-rural income gap (q = 0.512). The *q* value of the road network density (X7) was 0.397, and its explanatory power for the urban-rural income disparity was 39.7%. The *q* value of the per capita GDP index (X1) was 0.371, and its explanatory power for the urban-rural income disparity was 37.1%. The *q* values of the secondary industry output value in GDP (X3) and per capita fixed asset investment (X5) are also large with explanatory power reaching 29.9% and 27.1%, respectively, indicating that secondary industry development and regional development policies help to narrow the regional urban-rural income disparity.

Regarding the natural factors, topographic conditions and resource endowments have great explanatory power for the spatial differentiation of the urban-rural income gap, and the p values were all less than 0.01. The elevation (X10) and RDLS (X11) have the greatest power at explaining the spatial differentiation pattern of the urban-rural income gap, reaching 53.2% and 35.1%, respectively. The *q* value of NPP (X12) was 0.234, and the explanatory power was 23.4%. The annual average precipitation (X8) has weak explanatory power for the urban-rural income gap, but it can be combined with other factors to affect the spatial differentiation pattern of the urban-rural income gap.

Generally, the spatial differentiation pattern of the urban-rural income gap in the Yangtze River Economic Belt is affected by both natural factors and human activity factors. Socioeconomic factors are the dominant factors affecting the spatial differentiation of the urban-rural income gap, while natural environmental factors (temperature, topography and geological factors) are the secondary and basic factors affecting the urban-rural income gap.

#### Factors indicative effect analysis

In this study, the risk detector of the GeoDetector model was used to analyze the appropriate type or range of the influencing factors (Table 4), and a statistical significance test with a confidence level of 95% was conducted.

**Table 4:** The suitable type or range of factors.

Code	Factors	Suitable types or range
**X1**	Per capita GDP (yuan)	89589–156182
**X2**	Urbanization rate (%)	57.35–68.00
**X3**	The proportion of primary industry in GDP (%)	51.73–57.26
**X4**	The proportion of tertiary industry in GDP (%)	17.50–29.00
**X5**	Per capita fixed asset investment (yuan)	50303–66926
**X6**	Natural population growth rate (‰)	-0.84–3.32
**X7**	Road network density (km/km^2^)	1.51–1.759
**X8**	Annual mean precipitation (mm)	1643.45–1961.03
**X9**	Annual mean temperature (°C)	17.01–18.27
**X10**	Elevation (m)	1.29–77.96
**X11**	RDLS	0.003–0.3067
**X12**	NPP	855.76–1006.14

As shown in Table 4, regarding the socioeconomic factors, when the per capita GDP was 89589–156182 and the urbanization rate was 57.35–68.00%, the urban-rural income gap of the cities within this range was the smallest. This shows that the improvement of the economic development level and urbanization were conducive to narrowing the urban-rural income gap. When the proportion of primary industry in GDP is 51.73–57.26%, and the proportion of tertiary industry in GDP is 17.50–29%, the urban-rural income gap of the cities within this range is the smallest. When the per capita fixed asset investment was 50303–66926, the urban-rural income gap of the cities within this range was the smallest. Large-scale investment in fixed assets can improve the resource development intensity and market scale and then increase the income of urban and rural residents. When the natural population growth rate was -0.84–3.32‰, the urban-rural income gap in the cities within the index range was the smallest. When the road network density was 1.51–1.759, the urban-rural income gap of the cities within this range was the smallest.

Regarding the natural factors, the annual average precipitation, annual average temperature and NPP were 1643.45–1961.03mm, 17.01–18.27°C and 855.76–1006.14 respectively, and the urban-rural income gap was the smallest. When the elevation (1.29–77.96m) and RDLS (0–0.307) were the smallest, the urban-rural income gap was the smallest. These results show that there is a significant positive relationship between elevation, RDLS and the urban-rural income gap. The smaller the elevation and RDLS are, the smaller the urban-rural income gap is.

#### Analysis of the interaction between factors

**Fig 8:**
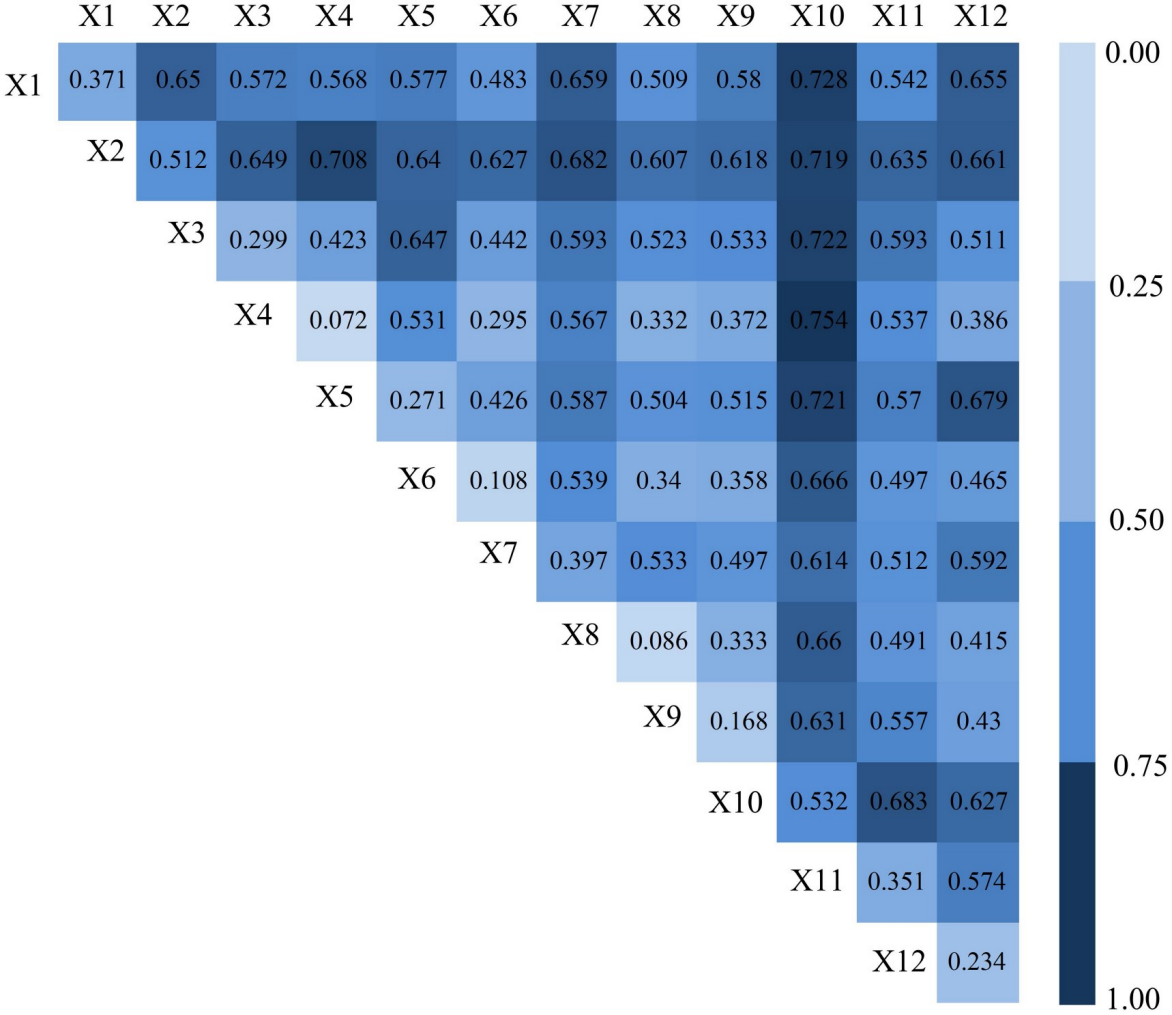
The *PD* values of the interaction between the factors.

The interaction detector can quantitatively identify the interactive influence of the interaction between two factors on the urban-rural income gap, that is, it can evaluate whether the joint action of factors X1 and X2 will increase or weaken the explanatory power of the dependent variable Y. The results of the analysis are shown in Fig 8, the *PD* values at positions (1,1), (2,2), (3,3), (4,4), (5,5), (6,6), (7,7), (8,8), (9,9), (10,10), (11,11), (12,12) represent the interactive influence of two same factors on dependent variables, while *PD* values at other positions represent the interactive influence of two different factors on dependent variables.

The results show that (Fig 8) the interactive influence (*PD* value) generated by the interaction between two factors was much higher than that of a single factor (*q* value), indicating that there is a close relationship between factors, and this has a significant interactive impact on the urban-rural income gap. Regarding the interaction between socioeconomic factors, PerGDP (X1), UR (X2), SIOP (X3), TIOP (X3), PerFAI (X5), NPGR (X6) greatly enhance the explanatory power of the spatial differentiation of the urban-rural income gap. Regarding the interaction of natural factors, the interaction of natural factors such as annual mean precipitation (X8), annual mean temperature (X9), RDLS (X11), NPP (X12) have greatly enhanced the explanatory power of the spatial differentiation of the urban-rural income gap. Regarding the interaction between socio-economic factors and natural factors, the explanatory power of the interaction between climatic factors (X8 and X9), topographic factors (X11) and economic development factors (X1), industrial structure factors (X3, X4) has been enhanced. The explanatory power of the interaction between topographic factors (X11) and population factors (X6) was also enhanced.

The factor interaction types include the mutual enhancement type and the nonlinear enhancement type. The proportion of two types was 48.5% and 51.5%, respectively. By comparing the *PD* value of the interaction with the sum of the two factors, we found that 52% of the PD value of the factor combination was greater than the sum of the *q* value of the factor combination, indicating that the interaction of factors greatly enhanced the influence on the urban-rural income gap. Overall, the effects of natural factors and socioeconomic factors on the urban-rural income gap are not independent of each other, but rather they interact with each other. The interaction of multiple factors on the urban-rural income gap is not a simple superposition process, but rather it is a mutual or nonlinear enhancement.

## Discussion

### Spatiotemporal pattern and driving force of urban-rural income gap

In this study, the coefficient of variation, Thiel decomposition index, spatial autocorrelation method and Geodetector model were used to analyze the spatial-temporal characteristics and driving forces of the urban-rural income gap in the Yangtze River Economic Belt from 2000 to 2017. The spatiotemporal analysis results show that the urban-rural income gap in the study area has obvious stage characteristics in the time series and significant spatial heterogeneity characteristics in the spatial distribution. Regarding the time series, the relative difference in the western region tends to expand, because the economic development in the western region is relatively backward, and the urban-rural income gap has not yet reached the peak of the inverted "U" shaped curve. The relative difference between the central and eastern regions shows the development trend of inverted an "U" shape, which is consistent with Kuznets’ inverted U-shaped curve theory. This shows that the evolution of the urban-rural income ratio in the central and eastern regions has obvious stage characteristics. In different stages, regional economic development has different influences on the urban-rural income gap. Regarding the spatial pattern, cities with higher and high income gaps were mainly distributed in western Sichuan Province, the Yunnan-Guizhou Plateau, western Hunan Province and northern Anhui Province, while cities with low and middle income gaps were mainly distributed in the middle reaches of three provinces and the Yangtze River Delta region, presenting an east-west differentiation pattern contrary to the economic development level.

Regarding the influencing mechanism of the factors, urbanization and economic development level can improve the wage income and property income of urban and rural residents; however, in the different stages of economic development, the income growth rate of urban and rural residents are not consistent, which is the direct cause of the disparity between urban and rural income in different regions. In this study, the urbanization rate and economic development level of the eastern and western regions show an east-west echelon distribution (eastern > central > western), which leads to the urban-rural income gap (eastern < central < western) showing an east-west differentiation pattern. The traffic factor is the basic condition of regional economic development. The greater the density of the road network is, the more conducive it is to the improvement of the rural economic development and income level. The development of secondary and tertiary industries can promote economic development and increase employment opportunities, thus affecting the urban-rural income gap through residents’ income.

Topographical conditions can limit the spatial distribution of population and economy, thus indirectly affecting the income of urban and rural residents (especially rural residents) through transportation, infrastructure construction and industrial development. The topographic conditions of the Yangtze Economic Belt show the characteristics of an east-west echelon distribution. Among the regions, the western region is located in a mountainous area. Its economic development is greatly restricted by the topography, and the overall urban-rural income gap is large. The central region is mostly located in the plain area, where economic development is less restricted by the topography, industry has a certain foundation, economic development is fast, and the urban-rural income gap is gradually narrowed. The eastern region is flat and economically developed, with a relatively small income gap. The natural resource endowment is an important resource support for regional socioeconomic development, which directly affects the urban-rural income gap.

### Comparison with previous studies

In this study, we used the Geodetector model to analyze the influences of socioeconomic factors and natural factors on the spatial pattern of the urban-rural income gap and explore the interactive influences of factors on the urban-rural income gap. The results showed that socioeconomic factors such as urbanization, the economic development level and industrial structure have significant impacts on the urban-rural income gap. This is consistent with the research results of Wang et al. [10], Guo et al. [14] and Zhang et al. [15]. Li [1] found that the secondary and tertiary industries of the Yangtze River Economic Belt accord with the Kuznets inverted U-shaped curve hypothesis, and the urban-rural income distribution changes with the process of industrial development. That is, as the the proportions of second and tertiary industries increase, the urban-rural income gap will expand, but when the industrial structure is upgraded to a certain stage, the urban-rural income gap will narrow again. This is consistent with our research results on industrial structure factors. Zhang et al. [22] found that favorable location conditions and convenient transportation have a significant impact on economic development and the reduction of urban-rural income gap. This is consistent with our findings. Regarding the natural factors, Jiang et al. [30] pointed out that topographic factors were the basic conditions for urban-rural construction and development, and they played a fundamental role in the spatial-temporal evolution of the urban-rural income gap. This is consistent with our findings.

### Scale effect

The scale effect of the urban-rural income gap. On the scale of the study area, the changing trend of the urban-rural income gap in the study area from 2000 to 2017 is consistent with Kuznets’ inverted U-shaped curve theory. On the regional scale, we find that the variation coefficient and Theil index in the central and eastern regions show an inverted "U"-shaped development process, which is consistent with Kuznets’ inverted U-shaped curve theory. However, the economic development in the western region is relatively backward, and the relative difference in the urban-rural income gap is still in the trend of fluctuating growth, which has not yet reached the peak of the inverted "U"-shaped curve. On the urban scale, the evolutionary trends of cities with different regions and different types of income gaps are also different (Fig 6).

Therefore, there are significant scale effects in the study of the urban-rural income gap. That is, under different scales, the research results will be different. On the regional scale and urban scale, we can find more problems, which is helpful to proposing regional development suggestions. It can be predicted that at a detailed research scale (counties, towns and villages), we can obtain more interesting findings.

### Limitations and future work

Most studies on influencing factors have focused on the relationship between single factors (economic growth [14], the industrial structure [15], industrialization [16], urbanization [17, 18], financial development [19], foreign trade [20], investment [20], fiscal expenditures [18], and labor mobility [21]) and the urban-rural income gap, but these studies rarely involve the comprehensive analysis of socioeconomic and natural factors, especially the natural environmental factors. On the basis of extensive reading of the literature, we construct a more comprehensive index system of influencing factors, and use the geographical detector model to analyze the impacts of socioeconomic factors and natural environmental factors on the income gap between urban and rural areas and the interaction between factors. Compared with other models, the Geodetector model has three unique advantages: First, the type of data is not limited, and the data can be numerical data or qualitative data [28]. Second, we can analyze the spatial heterogeneity of the influence of each influencing factor on the dependent variable. Third, we can detect the interaction between factors and the indicative effect of factors, and the analysis results are helpful to understanding the interaction mechanism between factors, and then promoting the sustainable development of society.

It is undeniable that our research still has the following two shortcomings: First, due to the complexity of the mechanism of socioeconomic factors and natural factors on the urban-rural income gap, the factors selected in this study may not be comprehensive enough. Therefore, in future research, we should expand more index types of influencing factors through new technical means, such as regional development strategy and policy factors, urban and rural factor circulation factors, and cultural factors. Second, there are extremely complex interactions among the influencing factors of the urban-rural income gap, so clarifying the complex interaction mechanism among factors plays an important role in further detailed studies of the urban-rural income gap. This is the direction that needs to be considered in future research.

## Conclusion

In this study, we used the coefficient of variation, Theil decomposition index, spatial autocorrelation method and GeoDetector model to analyze the temporal-spatial characteristics and influencing factors of the urban-rural income gap in the Yangtze River Economic Belt from 2000 to 2017. The main conclusions are as follows:

1. From 2000 to 2017, the per capita disposable income of urban and rural residents in the Yangtze River Economic Belt showed a trend of rapid growth. The relative differences in the study area show an inverted "U"-shaped development process, which is consistent with Kuznets’ inverted U-shaped curve hypothesis. Regarding the spatial pattern, the per capita disposable income of urban and rural residents in the study area showed a significant east-west differentiation pattern.
2. From 2000 to 2017, the urban-rural income gap in the study area showed an inverted "U"-shaped development process as a whole, and the relative difference showed an increasing trend. Regarding the different regions, the relative difference of the western regions showed an increasing trend while the relative differences in the central and eastern regions shows an inverted "U"-shaped development process as a whole, and there are obvious stage characteristics. Regarding the spatial pattern, the study area showed a significant east-west differentiation pattern. Regarding the spatial correlation effect, the spatial distribution of the urban-rural income gap in the study area has obvious positive spatial correlation, that is, the phenomena of high-value agglomeration and low-value agglomeration are significant.
3. The analysis of the influencing factors shows that the economic development level, the industrial structure, the regional development policy, transportation, topographical conditions and resource endowments can strongly explain the spatial differentiation pattern of the urban-rural income gap in the Yangtze River Economic Belt. The spatial differentiation pattern of the urban-rural income gap is affected by both natural factors and socioeconomic factors. Among the factors, socioeconomic factors are the dominant factors, and they are followed by natural factors. There is a significant interaction between natural factors and socioeconomic factors, and the combination of socio-economic factors and adverse natural factors can significantly affect the regional urban-rural income gap. The types of interaction among factors are mutual enhancement and nonlinear enhancement. The factor indicative effect analyzes the appropriate type or scope of influencing factors and proposes targeted regional development policies according to the appropriate type or scope of factors so as to promote the balanced development of urban and rural areas.

## Supporting information

S1 Table Regional differences in the per capita disposable income of urban residents. (PDF)

S2 Table Regional differences in the per capita disposable income of rural residents. (PDF)
