# Supplementary material for: Spatiotemporal differentiation of urban-rural income disparity and its driving force in the Yangtze River Economic Belt during 2000-2017
Source: PLoS One. 2021 Feb 4;16(2):e0245961. doi: 10.1371/journal.pone.0245961 (PMC7861366; doi:10.1371/journal.pone.0245961)
Supplement: S1 Table — (PDF) [file pone.0245961.s001.pdf]

S1 Table. Regional differences in the per capita disposable income of urban residents

Year	Variance coefficient				Theil index					
	S	West	Middle	East	S	Between-area	Within-area	West	Middle	East
2000	0.250	0.104	0.159	0.273	0.028	0.057	0.008	0.005	0.012	0.036
2001	0.253	0.106	0.162	0.275	0.029	0.058	0.008	0.005	0.012	0.036
2002	0.263	0.112	0.167	0.288	0.031	0.059	0.009	0.006	0.013	0.040
2003	0.263	0.118	0.171	0.287	0.031	0.058	0.009	0.007	0.014	0.040
2004	0.271	0.118	0.174	0.255	0.033	0.072	0.008	0.007	0.014	0.032
2005	0.259	0.114	0.164	0.252	0.030	0.067	0.008	0.006	0.013	0.031
2006	0.272	0.119	0.168	0.256	0.033	0.073	0.008	0.007	0.013	0.032
2007	0.276	0.123	0.154	0.275	0.034	0.071	0.009	0.007	0.011	0.036
2008	0.280	0.129	0.150	0.275	0.035	0.073	0.009	0.008	0.011	0.037
2009	0.288	0.138	0.144	0.276	0.037	0.076	0.009	0.009	0.010	0.037
2010	0.294	0.140	0.151	0.284	0.038	0.077	0.009	0.010	0.011	0.039
2011	0.309	0.141	0.150	0.298	0.042	0.081	0.010	0.009	0.011	0.043
2012	0.308	0.139	0.152	0.310	0.041	0.077	0.011	0.009	0.011	0.046
2013	0.297	0.151	0.145	0.318	0.039	0.069	0.011	0.011	0.010	0.048
2014	0.285	0.142	0.136	0.318	0.036	0.063	0.011	0.010	0.009	0.048
2015	0.273	0.135	0.132	0.322	0.033	0.056	0.011	0.009	0.008	0.050
2016	0.274	0.142	0.148	0.313	0.033	0.057	0.010	0.010	0.010	0.047
2017	0.262	0.166	0.147	0.292	0.031	0.054	0.010	0.013	0.010	0.041
