# Supplementary material for: Spatiotemporal differentiation of urban-rural income disparity and its driving force in the Yangtze River Economic Belt during 2000-2017
Source: PLoS One. 2021 Feb 4;16(2):e0245961. doi: 10.1371/journal.pone.0245961 (PMC7861366; doi:10.1371/journal.pone.0245961)
Supplement: S2 Table — (PDF) [file pone.0245961.s002.pdf]

S2 Table. Regional differences in the per capita disposable income of rural residents

Year	Variance coefficient				Theil index					
	S	West	Middle	East	S	Between-area	Within-area	West	Middle	East
2000	0.381	0.231	0.251	0.347	0.065	0.088	0.018	0.026	0.030	0.058
2001	0.385	0.234	0.259	0.348	0.067	0.088	0.018	0.026	0.032	0.059
2002	0.391	0.239	0.270	0.340	0.069	0.093	0.018	0.027	0.034	0.056
2003	0.402	0.221	0.276	0.341	0.073	0.096	0.018	0.024	0.036	0.056
2004	0.410	0.245	0.285	0.341	0.076	0.100	0.019	0.029	0.038	0.056
2005	0.424	0.258	0.286	0.346	0.081	0.107	0.020	0.032	0.040	0.058
2006	0.443	0.272	0.281	0.362	0.088	0.113	0.021	0.036	0.038	0.064
2007	0.459	0.280	0.273	0.369	0.094	0.119	0.022	0.038	0.036	0.066
2008	0.460	0.289	0.258	0.376	0.094	0.118	0.022	0.041	0.032	0.069
2009	0.471	0.309	0.248	0.384	0.099	0.121	0.023	0.047	0.030	0.072
2010	0.478	0.304	0.227	0.402	0.101	0.122	0.024	0.045	0.026	0.079
2011	0.494	0.319	0.227	0.413	0.107	0.125	0.025	0.049	0.026	0.084
2012	0.487	0.320	0.219	0.419	0.105	0.120	0.025	0.049	0.024	0.086
2013	0.469	0.315	0.209	0.400	0.098	0.116	0.023	0.048	0.022	0.078
2014	0.475	0.307	0.204	0.410	0.099	0.119	0.024	0.046	0.021	0.083
2015	0.457	0.311	0.197	0.377	0.093	0.118	0.021	0.047	0.020	0.069
2016	0.454	0.308	0.196	0.378	0.093	0.115	0.021	0.047	0.019	0.069
2017	0.439	0.304	0.195	0.362	0.088	0.110	0.020	0.046	0.019	0.064
